# Effects of vascular endothelial growth factor and angiopoietin-2 inhibitions on intraretinal hyperreflective foci in neovascular age-related macular degeneration

**DOI:** 10.1007/s10384-026-01392-7

**Published:** 2026-07-18

**Authors:** Yuta Nakanishi, Masahiro Akada, Ryosuke Tamiya, Midori Ideyama, Takahiro Kogo, Ai Ichioka, Akinari Yamamoto, Naoko Ueda-Arakawa, Hiroshi Tamura, Manabu Miyata, Ayako Takahashi, Ai Kido, Yuki Muraoka, Masahiro Miyake, Sotaro Ooto, Akitaka Tsujikawa, Masayuki Hata

**Affiliations:** 1 Department of Ophthalmology and Visual Sciences, Kyoto University Graduate School of Medicine, Sakyo-ku, Kyoto, 606-8507 Japan; 2 Center for Innovative Research and Education in Data Science, Institute for Liberal Arts and Sciences, Kyoto University, Kyoto, Japan

**Keywords:** Aflibercept, Anti-vascular endothelial growth factor, Faricimab, Hyperreflective foci, Neovascular age-related macular degeneration

## Abstract

**Purpose:**

To investigate the association of hyperreflective foci (HRFs) with disease activity in treatment-naïve neovascular age-related macular degeneration (nAMD) and to compare the effects of anti-vascular endothelial growth factor (VEGF) therapy versus dual VEGF-A/angiopoietin-2 (Ang-2) inhibition on HRFs.

**Study design:**

Retrospective, single-center, interventional study

**Methods:**

This study included 72 eyes with treatment-naïve nAMD. Thirty-two consecutive eyes received aflibercept 2mg and 40 consecutive eyes received faricimab, each undergoing three monthly loading injections. HRF presence and area, central retinal thickness (CRT), best-corrected visual acuity (BCVA), and associated parameters were evaluated at baseline and after loading. HRFs were quantified using binarized spectral-domain optical coherence tomography (SD-OCT) images with automated assistance.

**Results:**

At baseline, 45 eyes (62.5%) exhibited HRFs, all in the outer retina, with 10 eyes (13.9%) also showing inner retinal HRFs. Outer HRF area positively correlated with CRT and the maximum height of serous retinal detachment (SRD), while inner HRFs were associated with intraretinal fluid. Both treatments significantly improved BCVA and CRT. HRF presence and area decreased after treatment, primarily in the outer retina. After adjustment for baseline HRF area and clinical parameters, faricimab achieved a more substantial reduction in total, outer, and inner HRF areas than aflibercept.

**Conclusions:**

HRFs correlated with layer-specific exudative change. Faricimab resulted in a more pronounced reduction in HRFs than aflibercept despite comparable anatomical improvements, suggesting superior control of inflammation-related retinal changes.

**Supplementary Information:**

The online version contains supplementary material available at https://doi.org/10.1007/s10384-026-01392-7.

## Introduction

Neovascular age-related macular degeneration (nAMD) remains the leading cause of blindness worldwide despite the introduction of anti-vascular endothelial growth factor (VEGF) therapy [1]. Intravitreal injections of anti-VEGF drugs have shown to dramatically improve visual outcomes in patients with nAMD in the short term [2]. However, in some eyes, long-term vision outcomes remain poor even with continued anti-VEGF treatment because of fibrosis or macular atrophy development over time, gradual emergence of drug resistance, and suboptimal response to active anti-VEGF treatments. Thus, therapies beyond anti-VEGF are anticipated to increase therapeutic efficacy [3–5].

Intravitreal injections of faricimab (IVF), the first humanized, bispecific, IgG monoclonal antibody, which independently binds and neutralizes both angiopoietin-2 (Ang-2) and VEGF-A, enable the dual inhibition of two distinct pro-angiogenic signaling pathways involved in nAMD pathology [6, 7]. Neutralization of Ang-2 not only suppresses neovascularization but can also decrease vascular leakage and inflammation, and reduce the vascular responsiveness to VEGF-A, resulting in restoration of vascular stability [6]. Contrastingly, intravitreal injections of aflibercept (IVA) inhibit VEGF-A and other VEGF family members, including VEGF-B and placental growth factor (PlGF) [8]. The therapeutic response could vary between faricimab and aflibercept in patients with nAMD due to these pharmacological properties [9].

Chronic inflammation plays a pivotal role in the development of nAMD. Genomic and epidemiological studies have identified inflammation-related risk factors, such as gene mutations in *Complement factor H*, *C3*, *C4*, and *Tlr4*, aging, smoking, and obesity [10–12]. Chronic inflammation driven by innate immune activation, including age-related complement activation and monocyte infiltration into tissues, results in tissue damage and neovascularization [11, 13]. Given that subretinal fibrosis, one of the most significant nAMD treatment complications, is also caused by inflammatory responses, inflammation suppression is likely beneficial for nAMD activity control and could inhibit its complications [5, 14].

Hyperreflective foci (HRFs), well-circumscribed small dot structures showing high reflectivity noted in optical coherence tomography (OCT), are biomarkers of inflammatory retinal diseases, including diabetic retinopathy and AMD [15–18]. Although HRF’s precise origin remains unclear, HRFs are indicative of the inflammatory process of neovascularization and could imply nAMD disease activity. This study investigated the association of HRFs with disease activity in nAMD using automated-assisted quantitative measurement, and further assessed the effects of anti-VEGF treatments, including the differential impact of aflibercept and faricimab on HRFs.

## Materials and methods

### Study design and setting

This single-center, retrospective, nonrandomized, interventional study of consecutive patients was performed in an institutional setting. The Institutional Review Board of Kyoto University approved this study (No. R0532). All the study procedures adhered to the tenets of the Declaration of Helsinki. Informed consent was not required for the retrospective review of patient medical records, according to the guidelines of our Institutional Review Board.

### Patients and study population

We retrospectively reviewed the medical records of patients and included consecutive patients who were diagnosed with nAMD and received IVA between December 1, 2012, and October 31, 2013 (defined as the IVA group), and those who received IVF between June 1, 2022, and April 30, 2023 (defined as the IVF group). Of note, during the earlier period, all treatment-naïve patients with neovascular AMD were uniformly treated with aflibercept at Kyoto University Hospital. Similarly, during the later period, following the approval and reimbursement of faricimab in Japan, all treatment-naïve patients were treated with faricimab. The inclusion criteria encompassed the following: age ≥ 50 years, axial length < 26.5 mm, and presence of treatment-naive nAMD. The exclusion criteria included the following: the presence of other retinal diseases such as angioid streaks, diabetic retinopathy, vitelliform macular dystrophy, retinal vein or artery occlusion, patients who dropped out, eyes with subretinal hemorrhage greater than two disc diameters, eyes with extensive hard exudates within the macular scan area, and eyes with unclear OCT images. To avoid inter-eye correlation, if both eyes of a patient were eligible for the study, only the right eye was included in the analysis

### Intervention and observation procedure

During the loading phase, all patients received three monthly aflibercept (2 mg) or faricimab (6 mg) injections. Clinical data were collected at baseline and at the visit closest to 1 month after the final loading-phase injection. Patients underwent comprehensive examinations including BCVA measurement in the standard manner [19], axial length measurement (IOL Master, Carl Zeiss Meditec), fundus photography, spectral-domain OCT (SD-OCT), fluorescein angiography, and indocyanine green angiography (ICGA) at baseline. Briefly, BCVA was measured using Landolt C-charts at 5 meters after subjective refraction was performed by a trained orthoptist. Additionally, BCVA measurements and SD-OCT were conducted at the follow-up visit. SD-OCT images were obtained using Spectralis (Spectralis Family Acquisition Module, version 5.7.5.0) and the Heidelberg Eye Explorer (version 1.8.6.0; Heidelberg Engineering).

The SD-OCT images included 13 raster scans covering a 20 × 30-degree rectangular area with an average of 50 scans per session. Central retinal thickness (CRT) was defined as the distance between the vitreoretinal and inner surfaces of the RPE. Subfoveal choroidal thickness (SFCT) was measured using enhanced depth imaging scans, which included the distance between the outer border of Bruch’s membrane and the chorioscleral interface. CRT and SFCT were measured on both horizontal and vertical OCT B-scan images, and the average of two measurements were used for analysis. The maximum serous retinal detachment (SRD) height was measured among the 13 raster scans. MNV lesion size was measured on early- to mid-phase ICGA images using the area measurement tool integrated into the Spectralis system, which allows optical visualization of vascular lesions.

We assessed the presence of polypoidal lesions in each case at baseline using ICGA. Polypoidal choroidal vasculopathy (PCV) was diagnosed based on the characteristic polypoidal lesions at the border of the branching choroidal vascular networks. Eyes with signs of retinochoroidal anastomosis were diagnosed with retinal angiomatous proliferation (RAP). Eyes diagnosed with conditions other than PCV or RAP were diagnosed with typical AMD (tAMD).

### HRF measurements

We assessed the HRFs using the brightness of the RPE based on previously reported methods [17, 20, 21]. We processed the images on each of the 13 raster B-scans captured with Spectralis SD-OCT using ImageJ ver. 1.51 (Wayne Rasband, National Institutes of Health; http://rsb.info.nih.gov/ij/index.html). HRFs were defined as intraretinal hyperreflective dots with reflectivity equal to or greater than the mean brightness of the RPE. The average brightness of the RPE within a range of 1.5 mm and 3 mm from the foveal center was calculated in each B-scan image, and areas with reflectivity lower than the mean brightness of the RPE were automatically removed. If structures of the retinal nerve fiber layer and blood vessels remained, which sometimes had reflectivity comparable to that of the RPE, they were manually identified and removed. For HRF quantification, hard exudates were identified as yellow-white deposits on color fundus photographs corresponding to hyperreflective deposits on OCT B-scan images within the analyzed macular scan area, and these lesions were excluded from the HRF analysis. The remaining elements were classified as HRFs. The presence or absence of HRFs was independently evaluated by two retinal specialists (Y.N. and A.Y.), masked to treatment allocation (IVA or IVF). Any discrepancies were resolved through discussion with a senior specialist (H.M.). Based on their anatomical location, HRFs were further categorized as inner or outer retinal: the inner retina (defined as the layer between the internal limiting membrane and the inner nuclear layer) and the outer retina (defined as the layer between the outer plexiform layer and the RPE layer). The total area of the HRF was measured using ImageJ across 13 scans and was reported as µm^2^ per eye. All HRF and MNV areas were corrected for ocular magnification using the Bennett formula:　q = 0.01306 × (AL – 1.82), where AL represents the axial length [22].

### Statistical analysis

The BCVA was converted to the logarithm of the minimal angle of resolution. Descriptive statistics involved calculating the mean and standard deviation for continuous variables and proportions for categorical variables. All data were analyzed using JMP Statistics version 17.0 Pro (JMP Statistical Discovery LLC). Statistical significance was set at P < 0.05. We utilized a paired t-test or McNemar test to compare continuous or categorical variables between baseline and after the loading phase within the same eyes. We utilized the Wilcoxon rank-sum test or chi-square test as appropriate to compare baseline characteristics between independent groups, such as IVA vs. IVF, eyes with vs. without HRFs at baseline, or eyes with vs. without intraretinal fluid. We utilized Spearman’s rank correlation coefficients to examine the associations between HRF areas and parameters including BCVA, CRT, and maximum SRD height at baseline. For between-treatment comparisons of post-treatment HRF area, to evaluate between-group differences while accounting for baseline imbalances, we performed analysis of covariance (ANCOVA) with treatment group as a fixed effect and baseline HRF area, age, and sex as covariates, conducted separately for the whole, inner, and outer retinal layers.

## Results

### Baseline HRFs correlate with disease activity in treatment-naïve nAMD

Seventy-two eyes of 72 patients were included for this study; 32 and 40 eyes were treated with aflibercept (IVA group) and faricimab (IVF group), respectively. Among the 72 eyes, 32 exhibited tAMD, 36 demonstrated PCV, and four exhibited RAP. Table 1 shows the baseline characteristics of the patients. The mean age of the cohort was 75.6 ± 7.5 years. Of the 72 eyes, 67 (93.1%) exhibited subretinal fluid, and 17 (23.6%) demonstrated intraretinal fluid.

**Table 1:** Baseline clinical characteristics

Parameter	Total (n = 72)
Age (years)	75.6 ± 7.5
Sex (male/female)	44/28
Axial length, mm	23.51 ± 1.00
AMD subtypes tAMD/PCV /RAP	32/36/4
Visual acuity (LogMAR)	0.29 ± 0.31
MNV size µm^2^	2.7±2.4
CRT, µm	283.7 ± 119.5
Maximum SRD height, µm	143.1±102.2
Subretinal fluid (%)	67 (93.1)
Intraretinal fluid (%)	17 (23.6)
SFCT, µm	240.6 ± 97.8

Data are presented as means ± standard deviations where applicable

tAMD typical age-related macular degeneration; PCV polypoidal choroidal vasculopathy; RAP retinal angiomatous proliferation; MNV macular neovascularization; CRT central retinal thickness; SRD serous retinal detachment; SFCT subfoveal choroidal thickness

At baseline, 45 eyes (62.5%) exhibited HRFs within the retina, with all 45 eyes exhibiting HRFs in the outer retina (defined as outer retinal HRFs) and 10 eyes (13.9%) demonstrating HRFs in the inner retina (defined as inner retinal HRFs) (Supplementary Fig. 1a). The mean HRF area was 1155.3 ± 1871.6 µm^2^ in the total retina, 77.9 ± 247.4 µm^2^ in the inner retina, and 1077.4 ± 1794.8 µm^2^ in the outer retina (Supplementary Fig.1b). Eyes with outer retinal HRFs revealed a greater height of maximum SRD and tended to demonstrate a thicker CRT at the baseline than those without outer retinal HRFs (*P* = 0.011 and *P* = 0.058, respectively; Table 2).

**Table 2:** Baseline clinical characteristics between HRF positive and negative

Parameter	Outer retinal HRF			Inner retinal HRF		
	(+) 45eyes	(-) 27eyes	*P* value	(+) 10 eyes	(-) 62 eyes	*P* value
CRT, µm	303.8±121.4	250.22±110.3	0.058	314.2±126.2	278.8±118.7	0.313
Maximum SRD height, µm	170.0±113.6	98.4±57.9	0.011	157.6±113.7	140.8±101.0	0.757
Intraretinal fluid (%)	12 (26.7)	5 (18.5)	0.570	6 (60.0)	11 (15.3)	0.009
MNV size, µm^2^	2.8±2.1	2.5±2.8	0.217	2.4±1.3	2.7±2.5	0.705
Visual Acuity (LogMAR)	0.32±0.32	0.25±0.29	0.326	0.33±0.37	0.29±0.30	0.819

Data are presented as means ± standard deviations where applicable

*P* values for continuous variables were calculated using the Wilcoxon rank-sum test, and those for categorical variables were calculated using the chi-square test

CRT central retinal thickness; SRD serous retinal detachment; MNV macular neovascularization

**Fig. 1:**
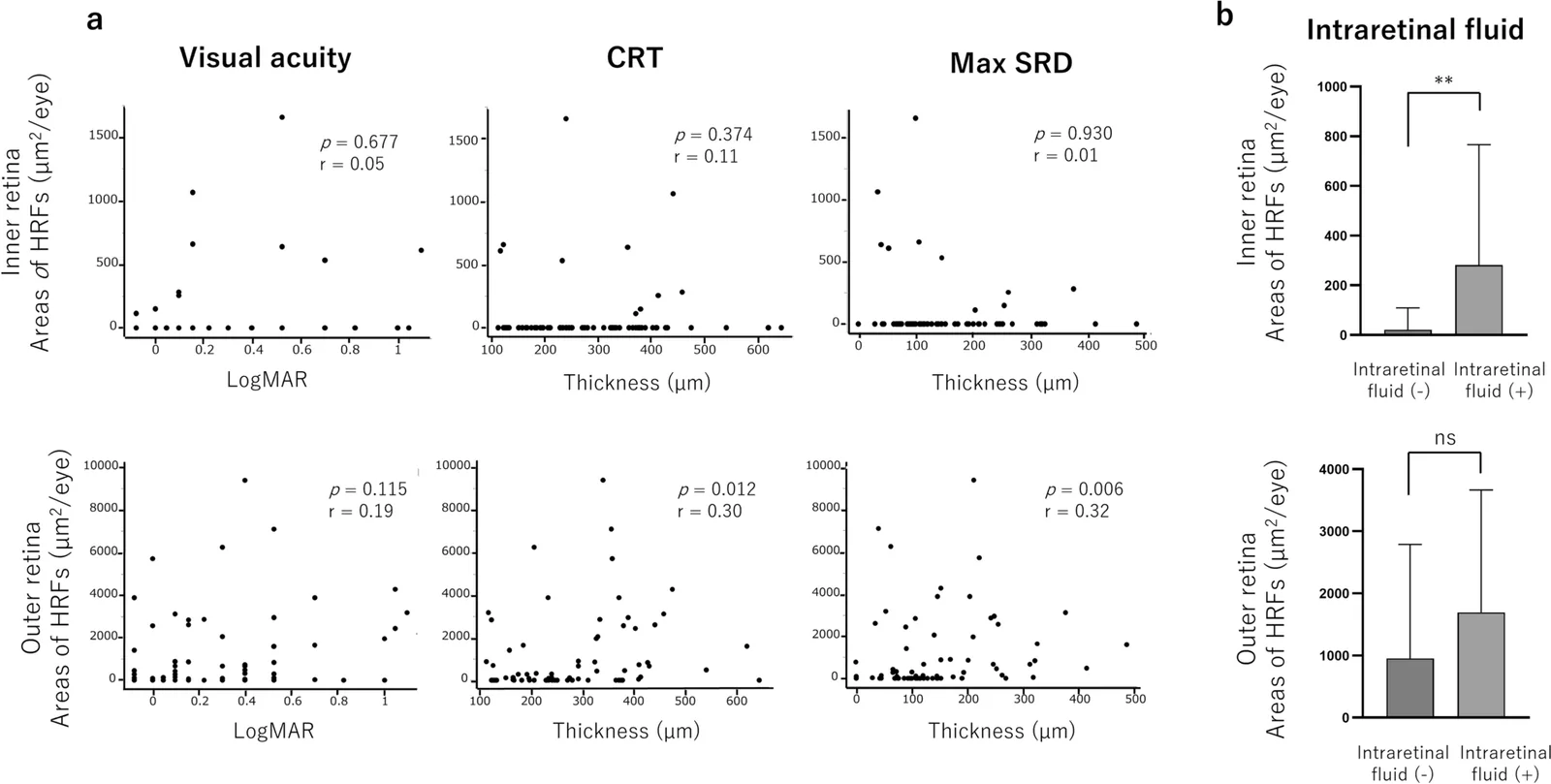
Associations between hyperreflective foci (HRF) areas and clinical parameters in neovascular age-related macular degeneration (nAMD). **a** Correlations between areas of HRFs in the inner retina (top row) or outer retina (bottom row) with visual acuity, central retinal thickness (CRT), and maximum serous retinal detachment (SRD) height. **b** Comparison of HRF areas between eyes with and without intraretinal fluid

The areas of the outer retinal HRFs were positively correlated with CRT (*P* = 0.012, r = 0.30) and maximum SRD height (*P* = 0.006, r = 0.32) at baseline (Fig. 1a). Contrastingly, eyes with inner retinal HRFs demonstrated intraretinal fluid more frequently than those without inner retinal HRFs (*P* = 0.009, Table 2). Conversely, eyes with intraretinal fluid exhibited greater areas of inner retinal HRFs but a similar extent of outer retinal HRFs compared to those without intraretinal fluid (*P* = 0.002, Fig. 1b). Collectively, the HRF presence and quantity are related to the severity of exudative changes in nAMD, and this association demonstrates layer specificity.

### Effects of anti-VEGF treatments on HRFs

After three monthly injections of either aflibercept or faricimab, logMAR BCVA, CRT, and SFCT significantly improved (all *P* < 0.0001, Table 3). Anti-VEGF treatments significantly reduced subretinal and intraretinal fluid (*P* < 0.0001 and *P* = 0.0045, respectively, Table 3). Additionally, anti-VEGF treatment reduced HRF presence and HRF area in the total retina (from 62.5% to 33.3%, *P* = 0.0002; from 1155.3 ± 1871.6 µm^2^ to 640.8 ± 1504.0 µm^2^, *P* = 0.0023, respectively), primarily in the outer retina (Table 3).

**Table 3:** clinical characteristics between baseline and after loading phase

Parameter	Baseline (n=72)	After loading phase (n=72)	*P* value
Visual acuity (LogMAR)	0.29±0.31	0.18±0.29	<.0001
CRT, µm	283.7±119.5	160.6±54.4	<.0001
SFCT, µm	240.6±97.8	222.4±93.2	<.0001
Subretinal fluid (%)	67 (93.1)	11 (15.3)	<.0001
Intraretinal fluid (%)	17 (23.6)	6 (8.3)	0.0045
Frequency of HRF (%)			
Total retina (%)	45 (62.5)	24 (33.3)	0.0002
Inner retina (%)	10 (13.9)	6 (8.3)	0.2482
Outer retina (%)	45 (62.5)	24 (33.3)	0.0002
Area of HRF (µm^2^)			
Total retina (µm^2^)	1155.3±1871.6	640.8±1504.0	0.0023
Inner retina (µm^2^)	77.9±247.4	49.1±235.8	0.2836
Outer retina (µm^2^)	1077.4±1794.8	591.7±1398.3	0.0019

Data are presented as means ± standard deviations where applicable

CRT central retinal thickness; SFCT subfoveal choroidal thickness; HRF hyperreflective foci

*P* values for continuous variables were calculated using the paired t-test, and those for categorical variables were calculated using the McNemar test

### Effects of aflibercept and faricimab treatments on HRF

**Fig. 2:**
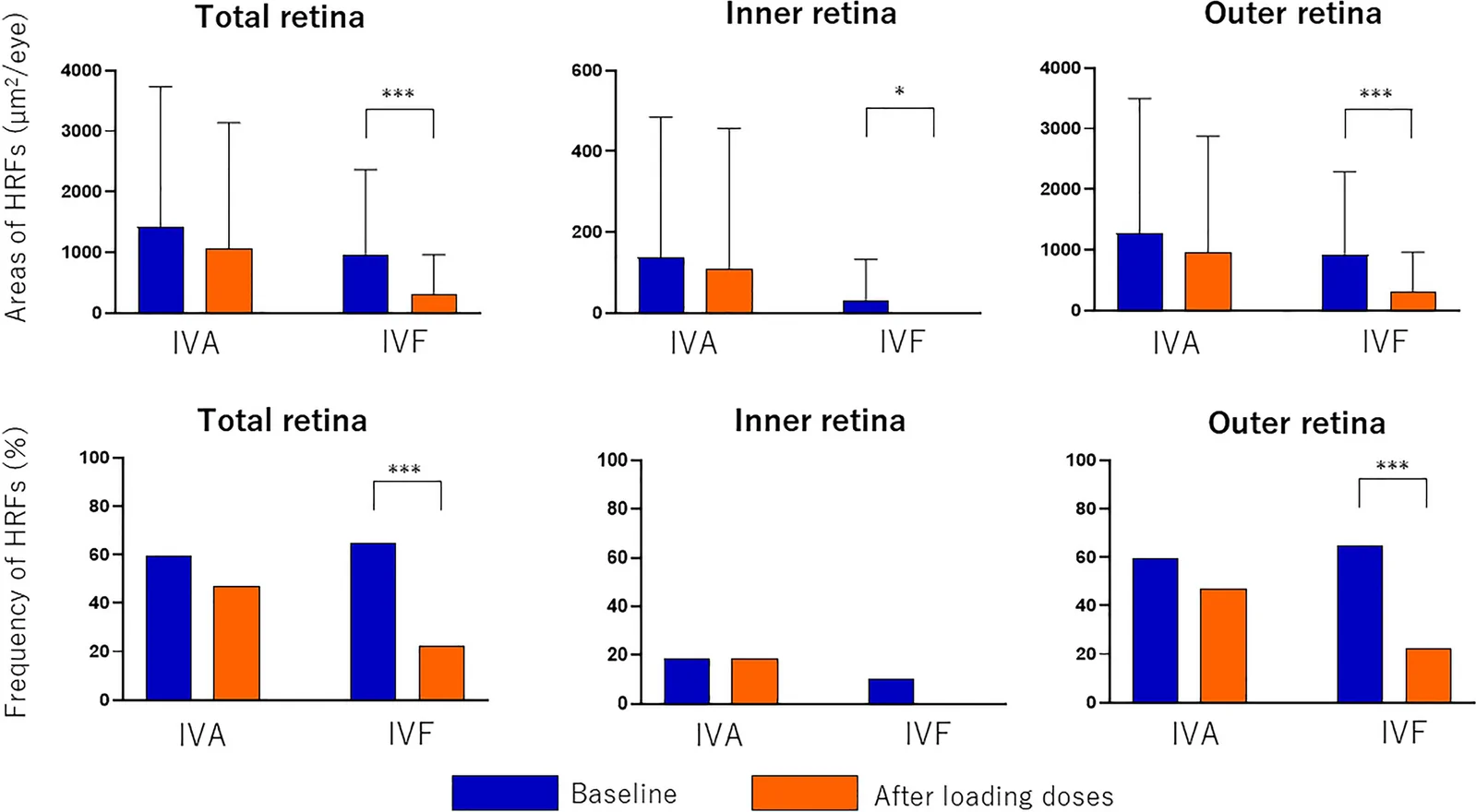
Changes in HRF areas and frequency before and after loading doses of aflibercept or faricimab.

The changes in logMAR BCVA, CRT, and SFCT after the loading phase did not differ significantly between the IVA and IVF groups (*P* = 0.73, 0.34, and 0.67, respectively; Supplementary Table 1). At baseline, the presence and areas of HRFs did not differ significantly between the aflibercept and faricimab groups (Fig. 2 and Supplementary Table 2). From baseline to after the loading phase, the presence of total retinal HRFs decreased from 59.4% to 46.9% in the aflibercept group, although this change was not statistically significant (*P* = 0.2482), whereas it significantly decreased from 65.0% to 22.5% in the faricimab group (*P* < 0.0001). Consistent with this finding, the total retinal HRF area significantly decreased in the faricimab group (*P* = 0.0002, Fig. 2). Faricimab also significantly decreased the areas of inner and outer retinal HRFs (*P* = 0.044 and 0.0002, respectively).

To further compare the treatment effects on HRFs while accounting for potential baseline imbalances, we performed analysis of covariance with post-treatment HRF area as the dependent variable, treatment group as the main explanatory variable, and age, sex, and baseline HRF area as covariates. After adjustment, treatment with IVF was significantly associated with smaller post-treatment HRF areas than treatment with IVA in the total retina, inner retina, and outer retina (total retina: β = 0.90, F = 5.60,* P* = 0.0209; inner retina: β = 0.50, F = 7.18, *P* = 0.0092; outer retina: β = 0.88, F = 5.60, *P* = 0.0210; Table 4).

**Table 4:** ANCOVA Analysis of the Effects of Clinical and Baseline Parameters on Post-treatment HRF Areas

	Total retina			Inner retina			Outer retina		
Covariates	β	F	*P* value	β	F	*P* value	β	F	*P* value
Age (years)	0.03	0.35	0.5534	0.01	0.37	0.5440	0.03	0.48	0.4896
Sex (male, female)	-0.01	0.00	0.9763	0.00	0.00	0.9867	0.02	0.00	0.9584
Baseline HRF areas (µm^2^)	0.34	9.71	0.0027	0.11	1.57	0.2148	0.36	11.20	0.0013
Drug (IVA, IVF)	0.90	5.60	0.0209	0.50	7.18	0.0092	0.88	5.60	0.0210

Analysis of covariance was performed with post-treatment HRF area as the dependent variable, treatment group as a fixed effect, and baseline HRF area, age, and sex as covariates. β indicates the regression coefficient, and F indicates the F statistic. IVA Intravitreal injection of aflibercept; IVF Intravitreal injection of faricimab

**Fig. 3:**
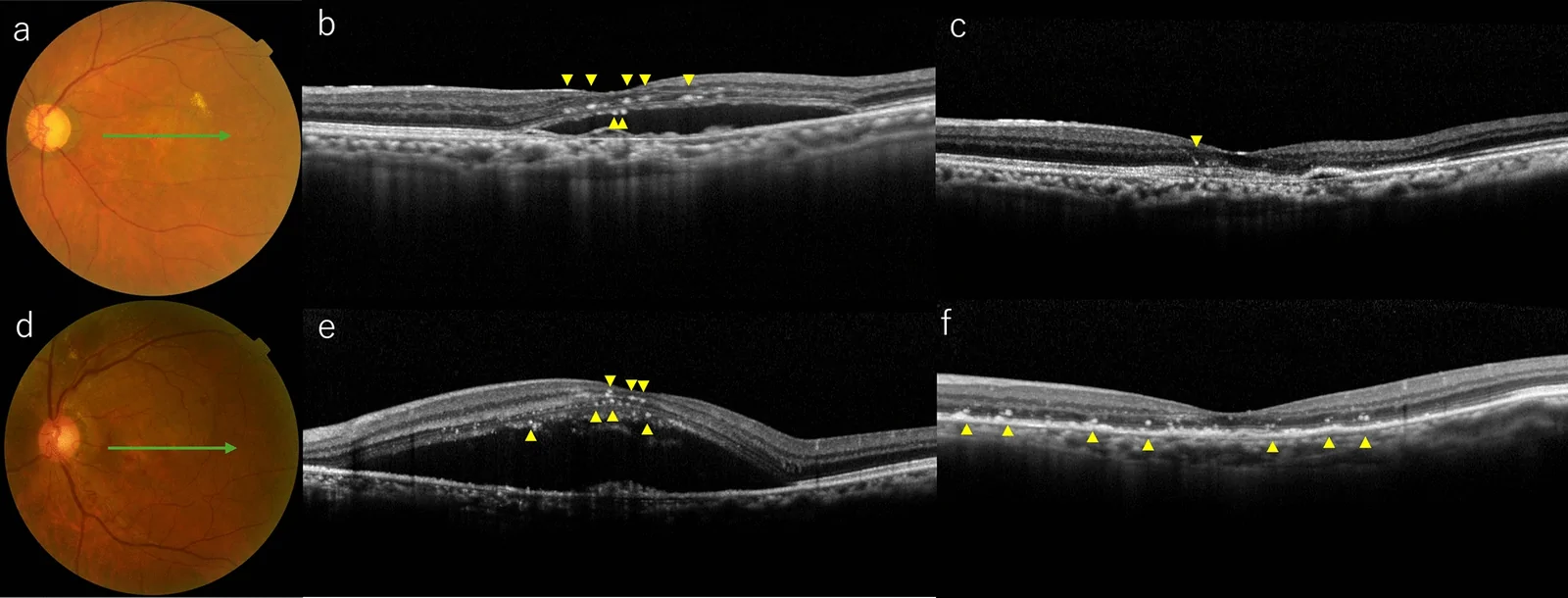
Representative cases of nAMD treated with faricimab (**a**–**c**) and aflibercept (**d**–**f**). **a**–**c** An 80-year-old patient treated with faricimab.** a** Fundus photograph shows retinal hemorrhage and hard exudates. **b** Baseline OCT image corresponding to the green arrow in **a** shows subretinal fluid, macular neovascularization (MNV), and multiple HRFs. **c** After loading doses of faricimab, the number of HRFs is reduced. **d**–**f** An 85-year-old patient treated with aflibercept. **d** Fundus photograph shows retinal hemorrhage. **e** Baseline OCT image corresponding to the green arrow in **d** shows subretinal fluid, MNV, and multiple HRFs. **f** After loading doses of aflibercept, HRFs persist. Yellow arrowheads indicate HRFs

Collectively, faricimab demonstrated more profound effects on HRFs than aflibercept, although both drugs similarly decreased retinal and choroidal thickness **(**Fig. 3**)**, suggesting that HRF modulation may involve drug-specific inflammatory pathways in addition to exudation control.

## Discussion

While the precise origin of HRFs remains elusive, several hypotheses have been proposed. These include the activated inflammatory cells such as microglia and infiltrating monocytes/macrophages within inflammatory environments, lipoprotein leakage, and the migration of RPE cells [16, 18, 23]. Lee et al. explored the relationship between soluble CD14 in the aqueous humor and HRF observed on OCT, suggesting that HRF may be indicative of activated microglia in Diabetic retinopathy patients [16]. Recently, in a study employing immunohistochemistry on donor eyes from patients with AMD some HRFs were revealed to be melanophages, showing positive staining for macrophage-specific markers and negative staining for RPE-specific markers. This indicates that some HRFs could be inflammatory cells such as microglia or macrophages that had phagocytosed melanin and melanolipofuscin [18].

HRFs are diffusely distributed throughout the retina in eyes with nAMD. The posterior eye segment contains various mononuclear phagocytes, including choroidal macrophages, retinal microglia, and perivascular resident macrophages [13], while circulating monocytes are additionally recruited during inflammation. Thus, in the context of retinal disease, distinct subsets of inflammatory cells are localized to specific retinal layers and may contribute to layer-specific formation of HRFs. Layer-specific distributions and clinical implications of HRFs are also reported in retinal vascular diseases. In diabetic macular edema, HRFs are frequently observed in the inner retina, whereas outer retinal HRFs are associated with photoreceptor layer disruption and visual dysfunction. In retinal vein occlusion, HRFs can be distributed across the affected retinal layers, and outer retinal HRFs have been associated with external limiting membrane disruption and poorer visual outcomes. These findings suggest that the layer-specific distribution and clinical significance of HRFs may differ according to the underlying disease mechanism [24–26]. In contrast, Christenbury et al. report that HRFs progressively migrate from the outer to the inner retinal layers in intermediate AMD, indicating that cells of common origin may demonstrate differential localization depending on their migration kinetics [20]. The current study reveals that the inner and outer retinal HRFs were independently related to disease activity in a layer-dependent manner. Additionally, anti-VEGF treatment led to a more pronounced reduction of HRF in the outer retinal layer in AMD, in line with previous studies [17]. This occurred despite the inner retinal layer being more directly exposed to the intravitreally injected anti-VEGF agents. The discrepancy in HRF dynamics and therapeutic effect between the inner and outer retinal layers suggest that HRFs in these layers may have distinct origins or pathological roles, providing a new perspective for further investigation.

Although both drugs similarly improved retinal and choroidal thickness, faricimab demonstrated a more pronounced effect on HRFs in nAMD than aflibercept. This difference may reflect the distinct pharmacological properties of faricimab, especially its dual inhibition of VEGF-A and Ang-2. Ang-2 plays a crucial role in retinal vascular diseases such as AMD, diabetic retinopathy, and retinal vein occlusion, by promoting vascular destabilization, increased vascular permeability, and pathological neovascularization along with VEGF-A [7, 27, 28]. Under pathological conditions, Ang-2 disrupts endothelial junction integrity and sensitizes the vasculature to VEGF-mediated angiogenic and inflammatory responses. Increased vascular permeability facilitates the extravasation of inflammatory cells into retinal tissue, thereby contributing to local inflammatory activation. In addition to its effects on vascular stability, Ang-2 can directly activate monocytes and macrophages, further amplifying inflammatory signaling, promoting inflammation. Simultaneous Ang-2 and VEGF-A inhibition reduces macrophage accumulation surrounding choroidal neovascularization in mouse models [6, 29]. Thus, the dual inhibition of Ang-2 and VEGF-A by faricimab may promote vascular stabilization while suppressing inflammatory cell recruitment which could explain the greater reduction in HRF quantity observed in the present study [30].

By contrast, aflibercept inhibits VEGF-A, VEGF-B and PlGF, known to contribute to angiogenesis and inflammation through VEGFR signaling [31]. However, aflibercept does not directly inhibit Ang-2–mediated vascular destabilization. The additional blockade of the Ang-2/Tie2 pathway by faricimab may, therefore, enhance endothelial barrier integrity and reduce vascular permeability, potentially limiting inflammatory cell infiltration and HRF formation. While no significant difference in the retinal thickness and visual function between aflibercept and faricimab over the short period, the greater anti-inflammatory effects and HRF reduction of faricimab may impact long-term outcomes in patients with nAMD, given that chronic inflammation is a primary driving force of the progression of pathological angiogenesis and retinal damages [11, 32]. Further studies are required to investigate the underlying mechanisms of Ang-2 inhibition on HRFs and their correlation with long-term clinical outcomes.

This study shows that the quantity of retinal HRFs fluctuates with nAMD activity, underscoring HRFs as a potential dynamic marker of disease activity. Outer retinal HRFs, observed in over 60% of treatment-naïve eyes, are associated with retinal thickness and the presence of SRD, a hallmark of type 1 MNV. Although progression to type 2 MNV with intraretinal fluid formation disrupts the external limiting membrane [33], this does not appear to increase outer retinal HRFs, suggesting their utility may be limited to earlier disease stages. In contrast, inner retinal HRFs are associated with intraretinal fluid, but a notable proportion of eyes with intraretinal fluid lacked inner retinal HRFs.

This study has several limitations. First, aflibercept and faricimab were administered nearly a decade apart due to drug availability and insurance. At Kyoto University Hospital, treatment-naïve nAMD patients are managed under standardized protocols, with all eligible patients receiving aflibercept in the earlier period and faricimab after its approval. Although the nationwide trends in nAMD may have shifted [34], baseline characteristics were comparable, SD-OCT was performed using the same system, and MNV activity was broadly equivalent, supporting the validity of our comparison. Second, we did not investigate the long-term effect of reduction in HRF. nAMD is a chronic disease that requires to be controlled for the long term, in which subretinal fibrosis formation and atrophy development can result in vision loss. Further long-term follow-up, with a focus on comparing IVA and IVF over an extended period, is required to understand the clinical significance of HRFs [5, 35]. Third, as we did not directly assess Ang-2 inhibition, the observed differences cannot be attributed solely to Ang-2 blockade. Variations in VEGF-A inhibition and aflibercept’s PlGF activity may have influenced HRF outcomes. Fourth, while we attributed the greater reduction in HRFs with faricimab to anti-inflammatory effects, the nature of HRFs in nAMD is unclear and may include RPE migration or lipid deposition, which OCT cannot distinguish. Thus, drug differences may involve mechanisms beyond inflammation, requiring validation with tissue-level or multimodal studies.

In conclusion, the quantity of HRFs fluctuates in correlation with nAMD activity and treatment efficacy, underscoring HRFs as potential dynamic markers of disease activity and therapeutic response in nAMD. The distinct effects of faricimab compared with aflibercept on HRF are likely due to its pronounced anti-inflammatory properties. Thus, combination therapies that target both angiogenesis and inflammation are crucial. Further studies with longer follow-up periods are required to assess how the varying effects of drugs on HRFs influence functional prognosis.

## Supplementary Information

Below is the link to the electronic supplementary material.

Supplementary file1 (TIF 611 KB)

Supplementary file2 (DOCX 19 KB)

Supplementary file3 (DOCX 17 KB)
