# Supplementary material for: Effects of vascular endothelial growth factor and angiopoietin-2 inhibitions on intraretinal hyperreflective foci in neovascular age-related macular degeneration
Source: Jpn J Ophthalmol. 2026 Jul 18;70(5):1165–73. doi: 10.1007/s10384-026-01392-7 (PMC13615986; doi:10.1007/s10384-026-01392-7)
Supplement: Supplementary file 1 [file 10384_2026_1392_MOESM2_ESM.docx]

| **Supplementary Table1　Clinical characteristics of baseline and after loading phase of aflibercept and faricimab** | | | | | | |
| --- | --- | --- | --- | --- | --- | --- |
| Parameter | IVA (n = 32) | | | IVF (n = 40) | | |
|  | Baseline | After loading phase | *P* value | Baseline | After loading phase | *P* value |
| Age (years) | 74.4 ± 7.6 | - | N.A. | 76.6 ± 7.4 | - | N.A. |
| Sex (male/female) | 18/14 | - | N.A. | 26/14 | - | N.A. |
| AMD subtypes tAMD/PCV /RAP | 12/17/3 | - | N.A. | 20/19/1 | - | N.A. |
| MNV size, mm^2^ | 2.2±2.4 | - | N.A. | 3.1±2.4 | - | N.A. |
| Visual acuity (LogMAR) | 0.28 ± 0.31 | 0.16 ± 0.29 | <.0001 | 0.30 ± 0.32 | 0.20 ± 0.29 | 0.0002 |
| CRT, µm | 285.8 ± 139.9 | 158.4 ± 28.1 | <.0001 | 282.0 ± 102.1 | 162.4 ± 68.9 | <.0001 |
| SFCT, µm | 250.6 ± 93.4 | 229.4 ± 92.9 | 0.0007 | 232.6 ± 101.6 | 216.7 ± 94.3 | 0.0034 |
| Maximum SRD height, µm | 128.1±113.2 | 12.3±29.6 | <.0001 | 155.2±92.1 | 15.8±39.5 | <.0001 |
| Data are presented as mean ± standard deviation or number, as appropriate. *P* values for comparisons between baseline and after the loading phase within each treatment group were calculated using the paired t-test. N.A., not applicable; IVA Intravitreal injection of aflibercept; IVF Intravitreal injection of faricimab; tAMD typical age-related macular degeneration; PCV polypoidal choroidal vasculopathy; RAP retinal angiomatous proliferation; MNV macular neovascularization; CRT central retinal thickness; SFCT subfoveal choroidal thickness; SRD serous retinal detachment. | | | | | | |
