# Supplementary material for: Effects of vascular endothelial growth factor and angiopoietin-2 inhibitions on intraretinal hyperreflective foci in neovascular age-related macular degeneration
Source: Jpn J Ophthalmol. 2026 Jul 18;70(5):1165–73. doi: 10.1007/s10384-026-01392-7 (PMC13615986; doi:10.1007/s10384-026-01392-7)
Supplement: Supplementary file 2 [file 10384_2026_1392_MOESM3_ESM.docx]

| **Supplementary Table 2**　 **Baseline HRF characteristics between the IVA and IVF groups** | | | |
| --- | --- | --- | --- |
| Parameter | IVA (n = 32) | IVF (n = 40) | *P* value |
| Frequency of HRF |  |  |  |
| Total retina (%) | 19 (59.4) | 26 (65.0) | 0.634 |
| Inner retina (%) | 6 (18.7) | 4 (10.0) | 0.323 |
| Outer retina (%) | 19 (59.4) | 26 (65.0) | 0.634 |
| Area of HRF |  |  |  |
| Total retina (µm^2^) | 1414.3±2317.7 | 948.1±1417.0 | 0.398 |
| Inner retina (µm^2^) | 138.3±346.3 | 29.5±103.5 | 0.055 |
| Outer retina (µm^2^) | 1276.0±2223.3 | 918.6±1370.0 | 0.597 |
| Data are presented as means ± standard deviations where applicable.  *P* values for the frequency of HRFs were calculated using the chi-square test, and *P* values for the area of HRFs were calculated using the Wilcoxon rank-sum test.  IVA Intravitreal injection of aflibercept; IVF Intravitreal injection of faricimab; HRF hyperreflective foci. | | | |
